# The diagnostic accuracy of the MTBDR*plus* and MTBDR*sl* assays for drug-resistant TB detection when performed on sputum and culture isolates

**DOI:** 10.1038/srep17850

**Published:** 2016-02-10

**Authors:** Michele Tomasicchio, Grant Theron, Elize Pietersen, Elizabeth Streicher, Danielle Stanley-Josephs, Paul van Helden, Rob Warren, Keertan Dheda

**Affiliations:** 1 Lung Infection and Immunity Unit, Division of Pulmonology and UCT Lung Institute, Department of Medicine, University of Cape Town, Cape Town, South Africa; 2 Department of Science and Technology/National Research Foundation Centre of Excellence for Biomedical Tuberculosis Research, South African Medical Research Council Centre for Tuberculosis Research, Division of Molecular Biology and Human Genetics, Faculty of Health Sciences, Stellenbosch University, Cape Town, South Africa; 3 Institute of Infectious Diseases and Molecular Medicine, University of Cape Town, Cape Town, South Africa

## Abstract

Although molecular tests for drug-resistant TB perform well on culture isolates, their accuracy using clinical samples, particularly from TB and HIV-endemic settings, requires clarification. The MTBDR*plus* and MTBDR*sl* line probe assays were evaluated in 181 sputum samples and 270 isolates from patients with culture-confirmed drug-sensitive-TB, MDR-TB, or XDR-TB. Phenotypic culture-based testing was the reference standard. Using sputum, the sensitivities for resistance was 97.7%, 95.4%, 58.9%, 61.6% for rifampicin, isoniazid, ofloxacin, and amikacin, respectively, whereas the specificities were 91.8%, 89%, 100%, and 100%, respectively. MTBDR*sl* sensitivity differed in smear-positive vs. smear-negative samples (79.2% vs. 20%, p < 0.0001 for ofloxacin; 72.9% vs. 37%, p = 0.0023 for amikacin) but not by HIV status. If used sequentially, MTBDR*plus* and MTBDR*sl* could rule-in XDR-TB in 78.5% (22/28) and 10.5% (2/19) of smear-positive and smear-negative samples, respectively. On culture isolates, the sensitivity for resistance to rifampicin, isoniazid, ofloxacin, and amikacin was 95.1%, 96.1%, 72.3% and 76.6%, respectively, whereas the specificities exceeded 96%. Using a sequential testing approach, rapid sputum-based diagnosis of fluoroquinolone or aminoglycoside-resistant TB is feasible only in smear-positive samples, where rule-in value is good. Further investigation is required in samples that test susceptible in order to rule-out second-line drug resistance.

Tuberculosis (TB) remains a leading cause of morbidity and mortality in developing countries^42^. National and global TB control efforts are undermined by the emergence of drug-resistant TB. MDR-TB is defined as resistance to rifampicin [RIF] and isoniazid [INH], and extensively drug-resistant TB (XDR-TB) is defined as MDR-TB plus resistance to a fluoroquinolone [FLQ] and a second-line injectable drug, such as amikacin [AMK], or capreomyin [CAP]^2^,^3^. DR-TB is associated with high mortality^4^,^5^,^6^,^7^, is a threat to healthcare workers^8^,^9^, and results in unsustainable costs that destabilise national TB control programmes (NTPs)^42^,^10^. If patients are placed on effective treatment earlier^11^,^12^,^13^, which can be facilitated by rapid genotypic rather than the slower phenotypic diagnostic testing, transmission will be reduced and the clinical prognosis of these patients will likely be improved.

In contrast to testing methods like the nitrate reductase assay^14^ and Microscopic Observation Drug Susceptibility (MODS)^15^, the MTBDR*plus* line probe assay (LPA; Hain Lifescience, Nehren, Germany), which is approved by the World Health Organisation (WHO) for the detection of RIF and INH resistance, is a same day test with a short relatively rapid within laboratory turn-around-time (~5 hours). It has an estimated sensitivity and specificity of 98.1% and 98.7% for both RIF and INH, when performed on culture isolates^16^, however, there are limited data about its performance using sputum. Several recent reports have reported a sensitivity of ~95% in smear-positive samples and ~65% in smear-negative samples^17^,^18^,^19^,^20^,^21^. Another LPA, MTBDR*sl*, was developed to diagnose XDR-TB by detecting mutations in the *gyr*A and *rrs* genes, thereby determining susceptibility to the FQs (ofloxacin, moxifloxacin, levofloxacin), and the second line injectable drugs (SLIDs; amikacin, kanamycin, and capreomycin). The assay displays a sensitivity and specificity of 85.1% and 98.2% to detect FQ resistance when performed directly (using smear-positive sputum samples)^22^, and 83.1% and 97.7, respectively when performed indirectly (using culture isolates)^22^. MTBDR*sl* exhibited sensitivities and specificities of 76.9% and 99.5% for the SLID class when performed indirectly (using isolates) and 94.4% and 98.2%, respectively when performed using smear-positive sputum samples (directly).

MTBDR*sl* makes the rapid same-day diagnosis of XDR-TB possible when it is used in combination with rapid tests for MDR-TB such as MTBDR*plus*. However, there are several gaps in our knowledge before such a strategy can be applied in appropriate settings. There are few data from a small number of cases about the performance of MTBDR*sl* using clinical samples^23^,^24^,^25^, none of which were smear-negative, and there are no studies evaluating the impact of HIV on accuracy. Moreover, a sequential testing strategy to inform clinical practice (e.g. MTBDR*plus* followed by MTBDR*sl*), and determinants of performance in this context, has hitherto not been evaluated. To address these considerations we evaluated the comparative diagnostic accuracy of the MTBDR*plus* and MTBDR*sl* assays using smear-positive and smear-negative sputum samples, and culture isolates obtained from patients in a TB and HIV endemic setting.

## Methods

### Study site and population

Sputum was collected from 234 patients enrolled in Brooklyn Chest Hospital, in Cape Town, South Africa, or undergoing routine testing at a centralised testing reference laboratory (National Health Laboratory Services [NHLS] at Groote Schuur Hospital). Brooklyn Chest Hospital is the designated provincial treatment centre for XDR-TB in the Western Cape. Brooklyn Chest Hospital also enrols patients with other types of drug-resistant TB. We accessed sputum or culture isolates from patients with culture-confirmed MDR-TB, or XDR-TB. The sputum samples and culture isolates came from different patients. Patients with confirmed drug susceptible isolates were sourced from the NHLS at Groote Schuur Hospital. DR-TB were based on phenotypical DST results. Patients were on anti-TB treatment at the time of specimen collection. In addition to the specimen collected for LPA testing, we collected a paired, second specimen that was used for microscopy and liquid culture. A HIV test was performed after appropriate counselling. This study was approved by the University of Cape Town Faculty of Health Sciences Ethics Committee and the methods were carried out in accordance with the approved guidelines. All patients provided written informed consent.

### Specimen processing

Samples were processed using the standard NALC-NaOH method (final NaOH concentration 1%^26^). Smear microscopy was performed using Ziehl-Neelsen staining. The WHO-recommended critical concentrations for RIF (1 μg/mL), INH (1 μg/mL), AMK (1 μg/mL) and ofloxacin (OFX) (2 μg/mL), were used for DST using the MGIT 960 liquid culture system (BD Bioscience, Erebodegem, Belgium^27^).

### MTBDR*plus* and MTBDR*sl* line probe assays

The MTBDR*plus* assay (version 1) and the MTBDR*sl* assay (version 1) were performed directly on a single sputum sediments. Sputum from 181 culture-positive patients received both tests. MTBDR*plus* and MTBDR*sl* were also performed indirectly on culture isolates (MTBDR*plus* and MTBDR*sl*; n = 270 received both tests) according to the manufacturer’s instructions (Hain Lifescience, Germany). The person performing the tests was blinded to the reference standard results. Manufacturer-recommended polymerase (HotStarTaq; Qiagen) was used for both LPAs, and the PCR on DNA from culture isolates was performed using the following parameters: 95 °C for 15 min, 95 °C for 30 s, 58 °C for 2 min (10 cycles), 95°C for 25 s, 53 °C for 40 s, 70 °C for 40 s (20 cycles) and final extension at 70 °C for 8 min (the parameters used for detecting DNA from sputum using the LPAs used 30 cycles of elongation). A valid LPA result was defined by a *Mycobacterium tuberculosis* complex-specific control (TUB), conjugate controls (CC) and amplification control (AC) bands in conjunction with the target gene locus control.

### Discrepant analysis

Sequencing was performed on the *inhA* promoter, *rpoB*, *katG*, *gyrA* and *rrs* genes, from isolates that were discrepant between phenotypic DST and either of the LPAs. The sequences of the primers used can be found in supplementary Table S1.

### Statistics

The sensitivity and specificity were calculated for each drug compared to the gold standard of culture-based DST. Patients whose paired sputum specimen was culture-negative were excluded. Statistical analyses were performed using Graphpad Prism (version 6.0; GraphPad Software, USA, www.graphpad.com), and STATA SE (version 12; StataCorp, USA). P-values less than 0.05 were considered significant. Fisher’s exact test with mid-P correction was used for comparisons between proportions.

## Results

### Patients and samples

The demographic data of the patients enrolled in the study is shown in Table 1. Demographic data was unavailable for 7/234 patients because of technical problems accessing the electronic NHLS records.

**Figure 1:**
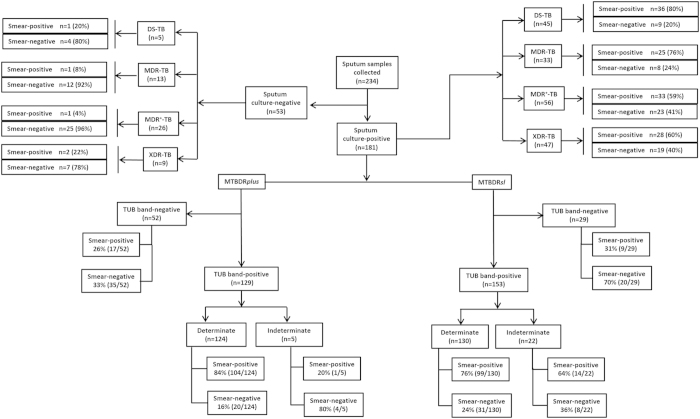
Study plan showing the number of sputum samples tested directly using MTBDR*plus* (version 1) or MTBDR*sl* (version 1) according to patients’ diagnoses and smear-status. The diagnosis was obtained using phenotypic liquid culture-based DST on a specimen collected at the same time as the specimen used for the line probe assays. A test is classified as positive for the *Mycobacterium tuberculosis complex* by the presence of the *M. tb* complex band (TUB), while a test is classified as negative by the absence of the *M. tb* complex band (TUB). Indeterminate results are those which are TUB-band positive yet are missing controls bands for gene specific loci. TB = tuberculosis; DS = drug sensitive, MDR = multi drug resistant, MDR+ =  MDR-TB but with additional resistance to OFX, KAN or INH. XDR = extensively drug resistant.

Figure 1 shows the study plan of the 234 patients tested using the LPAs. Fifty three patients were excluded because they were culture-negative. Of the 181 culture-positive samples, 45, 33, 56 and 47 were, according to phenotypic DST, DS-, MDR, MDR+ (MDR-TB and resistance to a FQ or SLID but did not meet the criteria for XDR-TB) and XDR, respectively.

### MTBDR*plus* performance

#### Direct testing of sputum samples by MTBDRplus

Accuracy: The diagnostic accuracy of MTBDR*plus* is shown in Table 2. The accuracy (sensitivity, specificity) for RIF^R^ and INH^R^ were (97.7%, 91.8%) and (95.4%, 89%), respectively. When the discrepant results were resolved by sequencing, the accuracy (sensitivity, specificity %) to detect RIF^R^ and INH^R^ increased to (100%, 100%) and (97.7%, 97.4%), respectively.

Indeterminate rate: The indeterminate rates are shown in Fig. 1. Amongst the MTBDR*plus*-TUB-positive sputum samples, 4% (5/129) were indeterminate. Twenty percent and 80% were smear-positive and smear-negative, respectively (p = 0.058).

Impact of HIV: Table 3 shows the sensitivities and specificities amongst samples from HIV-infected or -uninfected patients. The sensitivities and specificities to detect RIF^R^ or INH^R^ did not change according to HIV status.

#### Indirect testing of the culture isolates

Accuracy: The diagnostic accuracy of MTBDR*plus* for the culture isolates is shown in Table 4. The LPA had a sensitivity and specificity to detect RIF^R^ of 95.1% (95% CI 92.2% to 98.1%) and 100%, respectively and a sensitivity and specificity of 96.1% (93.5% to 98.7%) and 96.1% (90.8% to 100%), respectively to detect INH^R^.

Indeterminate rate: The study plan for the 270 culture isolates is shown in Figure S1. From the 270 culture isolates tested, 95.2% (257/270) were TUB band-positive and all were MTBDR*plu*s determinate. The indeterminate rate amongst the direct testing of the sputum samples (5/129) was significantly different to the indirect testing of the culture isolates (257/257; p < 0.001).

### MTBDR*sl* performance

#### Direct testing of sputum samples by MTBDRsl

Accuracy: The diagnostic accuracy for MTBDR*sl* is shown in Table 2. Overall the LPA exhibited suboptimal sensitivity for OFX^R^ (58.9% [95% CI 47.6% to 70.2%]) and AMK^R^ (61.6% [50.4% to 72.8%]). However, sensitivity was higher in smear-positive sputum samples (OFX^R^: 79.2% [95% CI 67.7% to 90.7%] and AMK^R^: 72.9% [60.3% to 85.5%], respectively; p = 0.473) compared to smear-negative sputum samples (OFX^R^: 20% [4.3% to 35.7%; p < 0.001] and AMK^R^: 37%; [18.8% to 55.2%; p < 0.001]), respectively. MTBDR*sl* displayed excellent specificities of 100% to detect OFX^R^ and AMK^R^ in sputum. Furthermore, the sensitivities and specificities of MTBDR*sl* to detect OFX^R^ and AMK^R^ did not significantly change when the discordant results were resolved by sequencing.

Impact of HIV: The diagnostic accuracy of MTBDR*sl* when stratified according to HIV status is shown in Table 3. Similarly to MTBDR*plus*, the sensitivities and specificities of MTBDR*sl* amongst the samples from HIV-infected versus HIV-uninfected patients were not significantly different at 48.1% versus 64.1% (p = 0.197) and 94.4% versus 83.3% (p = 0.26) for OFX^R^ and 59.2% versus 64.2% (p = 0.690) and 100% versus 90% (p = 0.166) for AMK^R^.

Indeterminate rate: Figure 1 shown that indeterminate rates for MTBDR*sl*. From the 153 TUB-positive sputum samples tested by MTBDR*sl*, 14.4% (22/153) were indeterminate, of which 64% (14/22) and 36% (8/22) were smear-positive and smear-negative, respectively (p = 0.070). The overall indeterminate rates are depicted in Table 2. Of the MTBDR*sl* TUB-positive results from smear-positive samples 1.6% (2/122) were indeterminate, compared to 5.1% (3/59) from smear-negative samples (p = 0.185).

#### Indirect testing of the culture isolates

Accuracy: The accuracy of MTBDR*sl* for the culture isolates is shown in Table 4. Indirect testing of the culture isolates by MTBDR*sl* showed a sensitivity and specificity of 72.3% (115/159) and 99% (100/101) for OFX^R^, respectively. For AMK^R^ the sensitivity and specificity was 76.6% (125/157) and 98% (99/101), respectively.

Indeterminate rate: The study plan of the culture isolates is shown in Figure S1. From the 270 culture isolates tested indirectly by MTBDR*sl*, 97% (262/270) were TUB band-positive and all were determinate. The proportion of indeterminate results for MTBDR*sl* between direct testing of sputum (14.4% [22/153]) and indirect testing of isolates (0% [262/262]; p < 0.001) was statistically different.

#### Comparison of direct and indirect testing for MTBDRsl

A comparison of the accuracy for the culture isolates versus the sputum samples using MTBDR*sl* is shown in Tables 4 and 2, respectively. MTBDR*sl* had increased sensitivity to detect OFX^R^ indirectly on the culture isolates (72.3% [115/159]) compared to direct testing of the sputum samples (58.9% [43/73]; p = 0.042). MTBDR*sl* had an improved sensitivity to detect AMK^R^ indirectly (76.6% [125/157] versus directly on sputum (61.6% [54/73]; p = 0.004).

### XDR-TB diagnosis by sequential use of MTBDR*plus* and MTBDR*sl* on sputum samples

**Figure 2:**
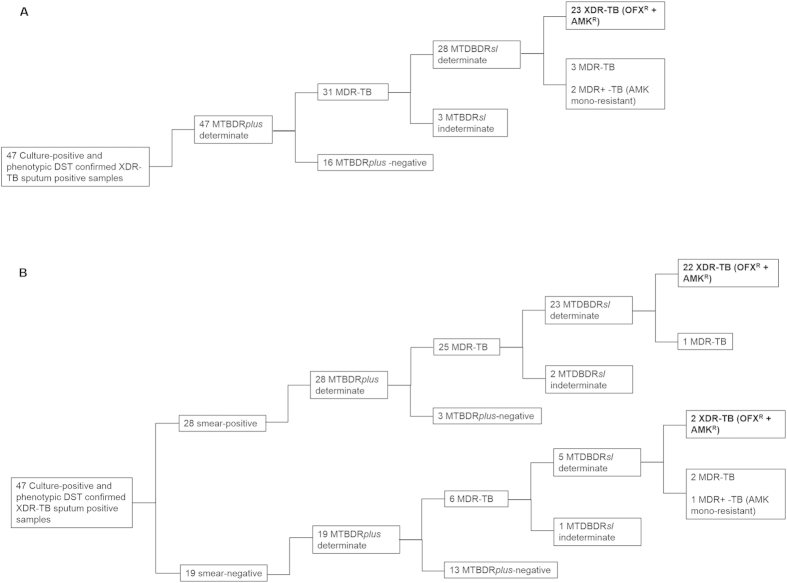
The testing pathway for the diagnosis of XDR-TB overall (**A**) or according to smear-status (**B**) in the clinical sputum specimens, when MTBDR*plus* and MTBDR*sl* were used sequentially. From the 234 sputum specimens tested, 47 culture-positive samples were diagnosed as XDR-TB by phenotypic DST.

OVERALL: Fig. 2A, depicts the ability of MTBDR*sl* to detect XDR-TB when performed directly and in conjunction with MTBDR*plus*. From the 47 culture-positive and phenotypically-confirmed XDR sputum samples, all 47 were determinate for MTBDR*plus* and 31 of these were detected as MDR-TB (RIF and INH resistant). Of these 31, 93.3% (28/30) were MTBDR*sl*-determinate and 23 were detected as XDR-TB (resistance to OFX and AMK). When used sequentially on sputum samples, MTBDR*plus* and MTBDR*sl* could thus rule in 49% (23/47 [95% CI 34.71% to 63.29%) of XDR-TB samples. The sequential use of MTBDR*plus* and MTBDR*sl* to detect XDR-TB when stratified according to smear-positive or smear-negative samples is shown in Fig. 2B. This testing strategy could rule in 78.5% (22/28) of smear-positive XDR-TB samples and 10.5% (2/19; p < 0.001) of smear-negative XDR-TB samples. The lower rule-in value in smear-negative samples is due to the high indeterminate rate relative to the smear-positive specimens.

## Discussion

We evaluated the diagnostic accuracy of MTBDR*plus* and MTBDR*sl* using sputum samples and culture isolates. There are hardly any data comparing the accuracy of MTBDR*sl* in smear-positive and smear-negative samples or interrogating the impact of HIV status. We show that MTBDR*plus* has excellent sensitivity for both RIF^R^ and INH^R^ using smear-positive and smear-negative sputum samples. By contrast, MTBDR*sl* showed modest sensitivity for OFX and AMK resistance in sputum samples. However, sensitivity was markedly reduced in smear-negative versus smear-positive sputum samples and indeterminate rates were elevated. Both LPAs had high specificity for the detection of drug-specific resistance, and thus a positive result for resistance can be treated with confidence.

This is the first study to evaluate the diagnostic accuracy of MTBDR*sl* directly on smear-negative clinical sputum samples. This information is critical to initiate rapid treatment and minimise transmission in areas with high HIV and TB co-infection where most patients are smear-negative^28^. Furthermore, although Xpert MTB/RIF can predict smear status^29^,^30^,^31^, the initial smear status of patients is often unknown and it can be unclear what DR-TB testing modality is suitable for sputum.

Sensitivities for OFX^R^ and AMK^R^ in sputum were lower than that published previously in our setting^32^, however, our study was the first to use smear-negative specimens. The reduced sensitivity of MTBDR*sl* amongst smear-negative samples indicate that, when used directly MTBDR*sl,* will likely only be useful in smear-positive sputum. The low sensitivity in smear-negative sputum can be explained by low concentrations of bacilli in the sputum, which are below the detection limit of the LPA^33^.

When performed indirectly on the culture isolates no MTBDR*plus* and MTBDR*sl*-TUB band positive results were indeterminate. However, when performed directly on the sputum samples, 4% of the MTBDR*plus*-TUB band positive results were indeterminate. By contrast, MTBDR*sl* had a high number of indeterminate results when performed directly at 14.4% (22/153). This is higher than has been reported in other studies^22^ and is explained by the testing of smear-negative samples, which harboured the bulk of the indeterminate (36%) readouts^34^.

This is the first study to evaluate a sequential testing strategy. The data is shown in Figs S2 and 2. We show that when MTBDR*plus* and MTBDR*sl* are used sequentially on DST culture-confirmed MDR+ or XDR-TB samples, the assays could rule-in 60%, 62.5% and 49% of OFX mono-resistant, AMK mono-resistant, and XDR-TB samples, respectively. When used sequentially on smear-positive XDR-TB samples, the assays could rule-in 78.6% of cases. However, the ability of the assay to accurately rule-in XDR-TB samples amongst the smear-negative sputum samples (10.5%) was substantially lower.

Overall our data indicate that MTBDR*sl* is likely a useful tool to rapidly diagnose MDR+ and XDR-TB, but only in smear-positive sputum samples. This is useful from a clinical and public health perspective as it enables a more rapid diagnosis (potentially by several weeks) thus likely minimising patient morbidity and mortality^32^, and most importantly ongoing transmission in the community. Transmission of DR-TB explains almost 80% of new cases in South Africa^35^ and has led to the emergence and transmission of resistance beyond XDR-TB^36^,^37^,^38^. We are of the view that the MTBDR*sl* assay should be used routinely in high MDR-TB burden programmatic settings in patients with rifampicin resistance. However, we acknowledge that further studies are urgently needed in different settings to confirm our findings so that global recommendations can be made that apply to high MDR-TB settings including South Africa and Eastern Europe. Our study represents the first step in this direction.

There are several limitations to our work. MTBDR*plus* version 1 and MTBDR*sl* version 1 were used, which have recently been succeeded by a new iteration (version 2)^39^,^40^. Nevertheless, diagnostic accuracy of MTBDR*plus* was excellent in the clinical sputum samples and similar to recent studies where version 2 of MTBDR*plus* was shown to have comparable accuracy to Xpert MTB/RIF in smear-negative sputum^39^,^41^. Both MTBDR*plus* and MTBDR*sl* were not performed at initial diagnosis, however, we collected a paired specimen for culture in order to control for the viability of the bacilli. Our samples size, particularly of the smear-negative group were small, yet substantially more than what has been reported elsewhere^21^,^22^,^23^,^24^,^25^. We also lacked data on the duration of treatment for each patient. A further limitation to the study was that both LPAs were only tested on culture-positive and not culture-negative sputum samples. There were 5 smear-positive culture-negative samples and these were excluded from the analysis given that they did not meet the reference standard definition. Finally, we did not test the impact of using MTBDR*plus* and MTBDR*sl* on treatment outcomes, cure, and death of the patients. However, our work lays the foundation for confirmatory and impact studies to now be undertaken.

In conclusion, even though MTBDR*sl* had suboptimal diagnostic sensitivity for OFX^R^ and AMK^R^, it remains an important rule-in tool to rapidly detect XDR-TB and MDR+ TB using smear-positive clinical samples, given that alternative tests have a prolonged within-laboratory turn-around-time and are technically challenging. Negative results, however, require further investigation as resistance to second line drugs may still be present but undetected by the assay. Smear-negative sputum specimens should be cultured prior to MTBDR*sl* testing.

## Additional Information

**How to cite this article**: Tomasicchio, M. *et al.* The diagnostic accuracy of the MTBDR*plus* and MTBDR*sl* assays for drug-resistant TB detection when performed on sputum and culture isolates. *Sci. Rep.* **6**, 17850; doi: 10.1038/srep17850 (2015).

## Supplementary Material

Supplementary Information

## Figures and Tables

**Table 1:** Demographic data of the cohorts used in study.

Demographic data	Study Cohort (%) (n = 227)*
Age	
Mean years (range)	37 (18–111)
Sex	
Male	110 (48)
Female	117 (52)
Race	
Black	109 (48)
Mixed	118 (52)
HIV-infected	
Yes	107 (47)
No	113 (50)
Unknown	7 (3)
CD4 count (cells/mL) range^Ψ^	308 (2–983)

^*^Demographic data for 7 patients was missing.

**Table 2:** Diagnostic accuracy of MTBDR*plus* and MTBDR*sl* for the direct detection of drug resistance in sputum samples using phenotypic culture-based susceptibility testing as a reference standard.

		All sputum samples		Smear-positive sputum		Smear-negative sputum	
		Sensitivity (%)	Specificity (%)	Sensitivity (%)	Specificity (%)	Sensitivity (%)	Specificity (%)
**MTBDR*****plus***‡**(v 1.0)**	RIF^R^*	97.7 (86/88)	91.8 (34/37)	97.1 (69/71)	91.2 (31/34)	100 (17/17) (p = 0.484)	100 (3/3)
	INH^R^*	95.4 (84/88)	89 (33/37)	95.6 (68/71)	88.2 (30/34)	94.1 (16/17) (p = 0.768)	100 (3/3)
**MTBDR*****sl***†**(v 1.0)**	OFX^R^	58.9 (43/73)	100 (38/38)	79.2 (38/48)	100 (34/34)	20 (5/25) (p < 0.001)	100 (4/4)
	AMK^R^	61.6 (45/73)	100 (38/38)	72.9 (35/48)	100 (34/34)	37 (10/27) (p < 0.001)	100 (4/4)

^‡^ 7.4% (9/122) MTBDR*plus* results from smear-positive samples were indeterminate, compared to 17% (10/59) from smear-negative (p = 0.049).

^†^1.6% (2/122) MTBDR*sl* results from smear-positive samples were indeterminate, compared to 5.1% (3/59) from smear-negative (p = 0.185). Refer to the materials and methods for a description of what defines an indeterminate result. P-values are for comparisons between smear statuses.

^*^When the discrepant results were resolved by sequencing the sensitivities and specificities of MTBDR*plus* were 100% and 100% to detect RIF^R^, respectively and 97.7% and 97.4% to detect INH^R^, respectively. RIF^R^ = rifampicin resistance, INH^R^  = isoniazid resistance, OFX^R^  = ofloxacin resistance, AMK^R^  = amikacin resistance.

**Table 3:** Diagnostic accuracy of MTBDR*plus* and MTBDR*sl* for the direct detection of drug resistance in sputum samples according to HIV status compared to phenotypic culture-based susceptibility testing (reference standard).

		All sputum samples				Smear-positive sputum				Smear-negative sputum			
		HIV-infected		HIV-uninfected		HIV-infected		HIV-uninfected		HIV-infected		HIV-uninfected	
		Sensitivity(%)	Specificity(%)	Sensitivity(%)	Specificity(%)	Sensitivity(%)	Specificity(%)	Sensitivity(%)	Specificity(%)	Sensitivity(%)	Specificity(%)	Sensitivity(%)	Specificity(%)
**MTBDR*****plus*****(v 1.0)**	RIF^R^	96.7 (29/30)	90 (9/10)	98 (49/50) (p = 0.712)	88.2 (15/17) (p = 0.888)	100 (23/23)	100 (8/8)	97.7 (43/44) (p = 0.466)	100 (14/14)	100 (7/7)	100 (1/1)	100 (8/8)	100 (1/1)
	INH^R^	93.3 (28/30)	90 (9/10)	96 (48/50) (p = 0.596)	88.2 (15/17) (p = 0.888)	91.6 (22/24)	100 (8/8)	97.6 (41/42) (p = 0.264)	50 (2/4)	100 (7/7)	100 (1/1)	87.5 (7/8) (p = 0.333)	100 (1/1)
**MTBDR*****sl*****(v 1.0)**	OFX^R^	48.1 (13/27)	94.4 (17/18)	64.1 (25/39) (p = 0.197)	83.3 (25/30) (p = 0.260)	69 (11/16)	100 (9/9)	82.1 (23/28) (p = 0.308)	100 (17/17)	18.2 (2/11)	100 (2/2)	18.2 (2/11)	100 (1/1)
	AMK^R^	59.2 (16/27)	100 (18/18)	64.1 (25/39) (p = 0.690)	90 (27/30) (p = 0.166)	62.5 (10/16)	100 (9/9)	78.6 (22/28) (p = 0.250)	100 (17/17)	50 (6/12)	100 (2/2)	27.3 (3/11) (p = 0.265)	100 (1/1)

P-values are for comparisons between HIV statuses.

RIF^R^ = rifampicin resistance, INH^R^ = isoniazid resistance, OFX^R^ = ofloxacin resistance, AMK^R^ = amikacin resistance.

**Table 4:** Diagnostic accuracy of MTBDR*plus* and MTBDR*sl* for the detection of drug resistance in culture isolates compared to phenotypic culture-based susceptibility testing (reference standard).

		Sensitivity (%)	Specificity (%)
**MTBDR*plus*(v 1.0)**	RIF^R^	95.1% (196/206) (p = 0.117)	100% (51/51) (p = 0.039)
	INH^R^	96.1% (198/206) (p = 0.495)	96.1% (49/51) (p = 0.698)
**MTBDR*****sl*** **(v 1.0)**	OFX^R^	72.3% (115/159) (p = 0.042)	99.0% (100/101) (p = 0.538)
	AMK^R^	76.6% (125/157) (p = 0.004)	98.0% (99/101) (p = 0.382)

P-values are for comparisons between direct testing on specimens (both smear-positive and smear-negative; data shown in Table 2).
