# Supplementary material for: The diagnostic accuracy of the MTBDRplus and MTBDRsl assays for drug-resistant TB detection when performed on sputum and culture isolates
Source: Sci Rep. 2016 Feb 10;6:17850. doi: 10.1038/srep17850 (PMC4748215; doi:10.1038/srep17850)
Supplement: Supplementary Information [file srep17850-s1.doc]

**SUPPLEMENTARY MATERIAL**

The diagnostic accuracy of the MTBDR*plus* and MTBDR*sl* assays for drug-resistant TB detection when performed on sputum and culture isolates.

Michele Tomasicchio^1,*^, Grant Theron^2^,^1,*^, Elize Pietersen^1^, Lizma Streicher^2^, Danielle Ruth Stanley^2^, Paul van Helden^2^, Rob Warren^2^, Keertan Dheda^1,3^

**Affiliations:**

^1^Lung Infection and Immunity Unit, Division of Pulmonology and UCT Lung Institute, Department of Medicine, University of Cape Town, Cape Town, South Africa.

^2^Department of Science and Technology/National Research Foundation Centre of Excellence for Biomedical Tuberculosis Research, South African Medical Research Council Centre for Tuberculosis Research, Division of Molecular Biology and Human Genetics, Faculty of Health Sciences, Stellenbosch University, Cape Town, South Africa.

^3^Institute of Infectious Diseases and Molecular Medicine, University of Cape Town, Cape Town, South Africa.

^*^Authors contributed equally.

**Correspondence:** Keertan Dheda, Lung Infection and Immunity Unit, Division of Pulmonology and UCT Lung Institute, Dept of Medicine, University of Cape Town, South Africa. E-mail: keertan.dheda@uct.ac.za

**S1. METHODS**

**Discrepant analysis.**

The sequences of the primers used in the study are shown in Table S1 and the sequencing methodology can be found in Streicher et al. [^38^](#_ENREF_38). If a phenotypically-susceptible specimen possessed non-synonymous mutations in the resistance determining regions of the *inhA* promoter, *rpoB*, *katG*, *gyrA* or *rrs* genes, it was classified as resistant.

**S2. RESULTS**

**Study plan.**

The study plan for the 270 culture isolates tested using the LPAs is shown in figure S1. Fifty five, 50, 79 and 86 of the culture isolates were classified as DS-, MDR-, MDR^+^- and XDR-TB, respectively by phenotypic DST.

**MTBDR*plus* performance outcomes**

*Direct testing of sputum samples by MTBDRplus*

ACCURACY: MTBDR*plus* was tested on 181 culture-positive sputum samples of which a total of 71.3% (129/181) tested positive for TB (TUB band-positive) (Figure 1). Twenty seven percent (35/129) and 73% (94/129) of the MTBDR*plus*-positive sediments were classified as DS- and MDR-TB, respectively.

The sensitivity of MTBDR*plus* to detect RIF^R^ was 97.1% (93.2% to 100%) for smear-positive and 100% for smear-negative samples (p=0.484). The sensitivity of MTBDR*plus* to detect INH^R^ in smear-positive sputum samples (95.6%; 90.83% to 100%) compared to smear-negative sputum samples (94.1%; 82.9% to 100%) did not differ (Table 2; p = 0.768). The RIF^R^ and INH^R^ discrepant results were resolved by sequencing the *rpo B* and *inh A* genes, respectively. Four (D516F, G531T, D516F, F511C) and one (mutation at base pair -15 relative to the transcriptional start site) of the clinical isolates were associated with RIF and INH resistance, respectively.

INDETERMINATE RATE: We show that from the 181 culture-positive sputum samples, 122 and 59 were smear-positive and smear-negative, respectively and 7.4% (9/122) of the MTBDR*plus* results from smear-positive samples were indeterminate, compared to 17% (10/59) from the smear-negative samples (Table 2, p=0.049).

**MTBDR*sl* performance outcomes**

*Direct testing of sputum samples by MTBDRsl*

IMPACT OF HIV: Within the smear-positive sputum samples the sensitivities of MTBDR*sl* to detect OFX^R^ or AMK^R^ did not differ in the HIV infected versus HIV uninfected samples (Table 3; 69% vs. 82.1% for OFX^R^, respectively; p=0.308 and 62.5% vs. 78.6 for AMK^R^, respectively; p=0.250). However, within the smear-positive sputum samples there appeared to be a decrease in the sensitivity for OFX^R^ amongst the HIV-infected versus the HIV-uninfected group (69% versus 82.1%, respectively), but this was not significant (p=0.308). The specificities of the LPA for OFX^R^ and AMK^R^ remained at 100% for the HIV infected and HIV uninfected samples within the smear-positive and smear-negative groups (Table 3).

**MDR+-TB diagnosis by sequential testing using of MTBDR*plus* and MTBDR*sl* on sputum samples**

We examined the ability of MTBDR*plus* and MTBDR*sl* when used sequentially to diagnose MDR+-TB directly from clinical sputum samples. When used sequentially, MTBDR*plus* and MTBDR*sl* could rule-in 60% (18/30 [CI 95% 42.5% to 77.5%]) and 62.5% (15/24 [43.1% to 81.9%]) of OFX and AMK mono-resistance samples, respectively (Figure S2). Within the 30 OFX and 24 AMK mono-resistant culture-positive DST confirmed samples, 10% (3/30) and 8.3% (2/24) were initially indeterminate by MTBDR*plus*, respectively. Of the 27 and 22 MTBDR*plus* determinate OFX and AMK mono-resistant culture-positive DST confirmed samples, respectively, 66.6% (18/27) and 72.7% (16/22) were positive for MTBDR*plus*, respectively and all of the MTBDR*plus* positive samples were MTBDR*sl* determinate.

**MDR+ and XDR-TB diagnosis of the culture isolates indirectly by sequential use of MTBDR*plus* and MTBDR*sl***

We tested the sequential ability of MTBDR*plus* and MTBDR*sl* to detect MDR+ and XDR-TB samples indirectly in the culture isolates. From the 36 and 43 isolates determined as mono-resistant (to either OFX or AMK, respectively), when used sequentially MTBDR*plus* and MTBDR*sl* could rule-in 66.6% (24/36) and 74.4% (32/43) of the OFX and AMK mono-resistant samples, respectively (Figure S3 A and B). Similarly, when used sequentially, MTBDR*plus* and MTBDR*sl* could rule-in 81% (70/86) of all XDR-TB samples (Figure S3 C).

**Tables.**

**Table S1. Primers used in the current study to resolve the discordant results.**

| **Primer** | **Sequence (5’-3’)** |
| --- | --- |
| ***katG***  RTB For  RTB Rev | TGGCCGCGGCGGTCGACATT  GGTCAGTGGCCAGCATCGTC |
| ***inhA* promoter**  inhA For  inhA Rev | CGCAGCCAGGGCCTCGCTG  CTCCGGTAACCAGGACTGA |
| ***rpoB***  rpoB For  rpoB Rev | TGGTCCGCTTGCACGAGGGTCAGA  CTCAGGGGTTTCGATCGGGCACAT |
| ***gyrA***  GyrA For  GyrA Rev | TGACATCGAGCAGGAGATGC  GGGCTTCGGTGTACCTCATC |
| ***rrs 1400 region***  rrs290 For  rrs290 Rev | TGCTACAATGGCCGGTACAA  CTTCCGGTACGGCTACCTTG |

**Figures.**

Figure S1.

**
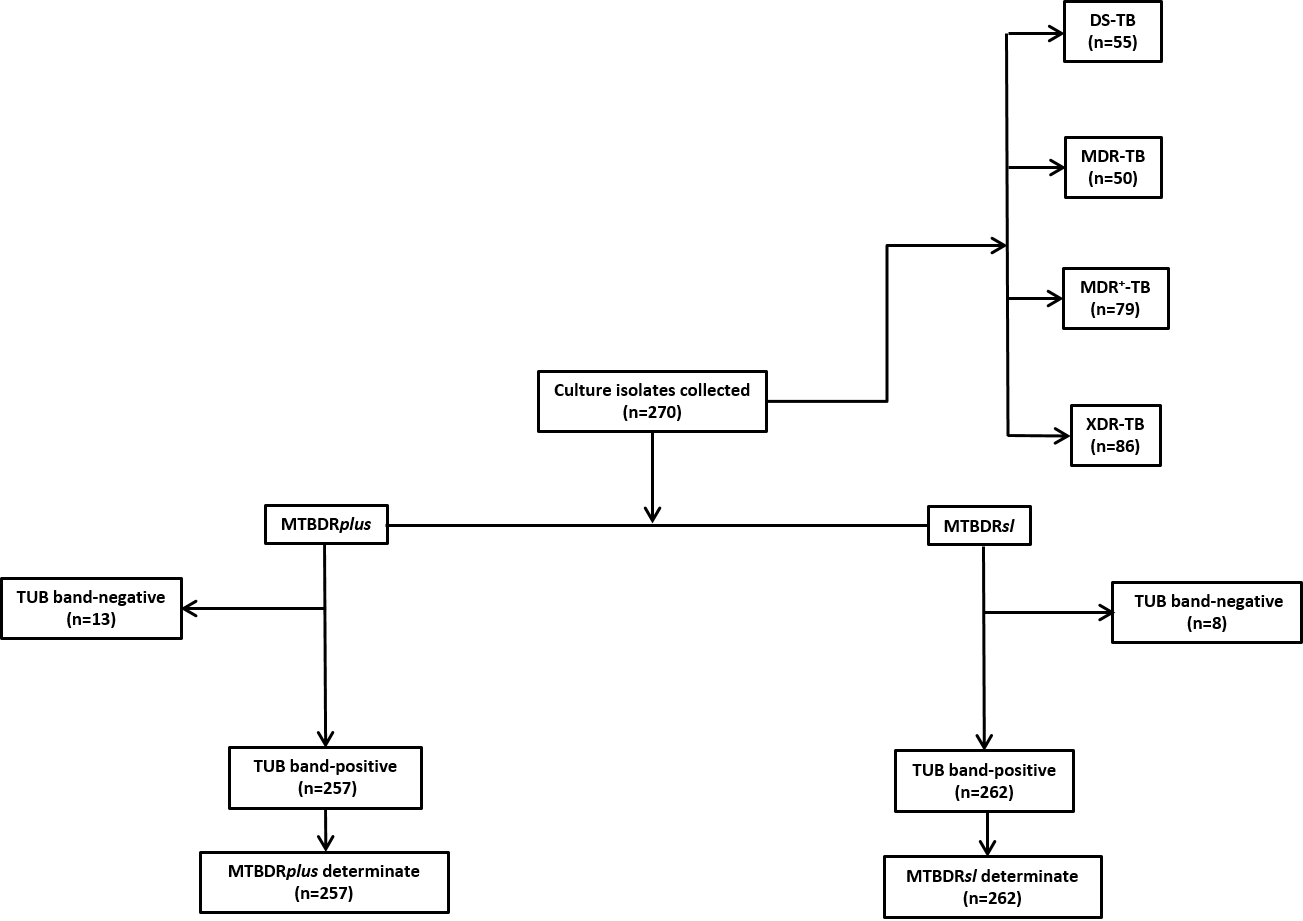
**

Figure S2.


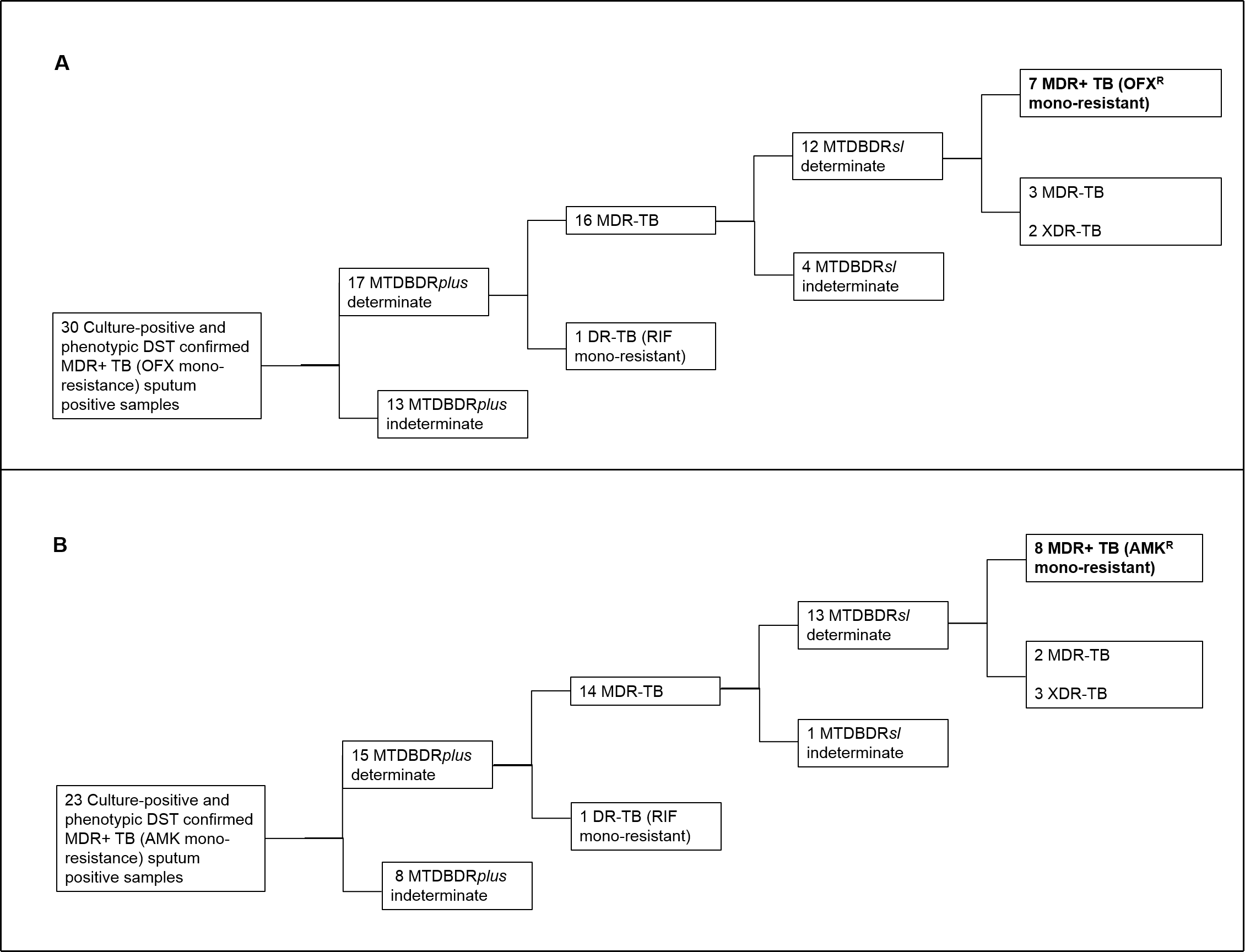


Figure S3.


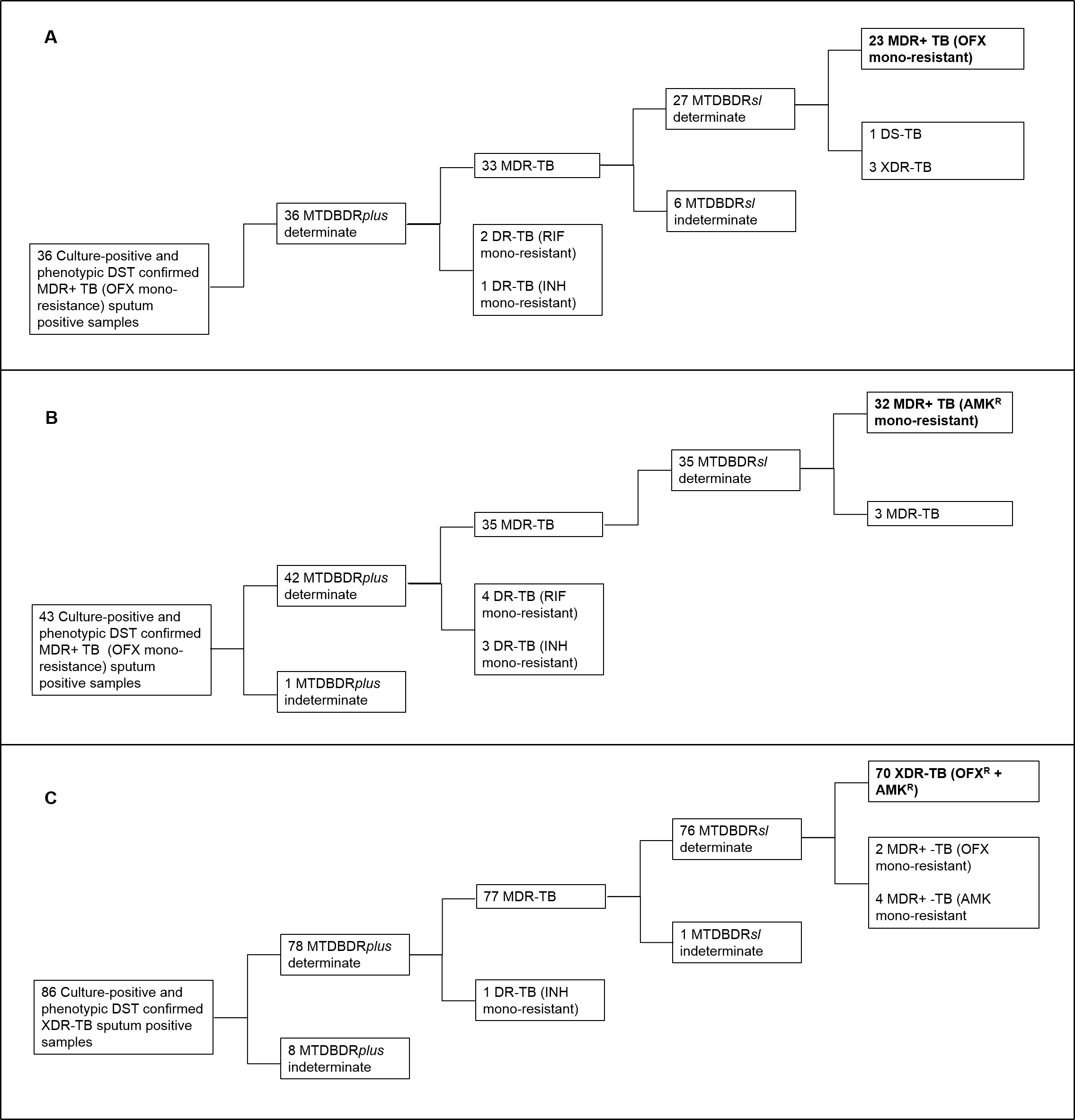


**Figure Legends.**

**Figure S1:** The study plan for 270 culture isolates tested indirectly using MTBDR*plus* and MTBDR*sl*. Phenotypic DST served as the reference standard for DS-, MDR-, MDR^+^- and XDR-TB. The LPA is considered positive for *Mycobacterium tuberculosis* by the presence of the *M. tb* TUB band and indeterminate if TUB band-positive but missing a control band for the gene specific loci

**Figure S2:** Diagrammatic representation for the diagnosis of MDR+-TB, defined as OFX mono-resistant (**A**) or AMK mono-resistant (**B**) in the clinical sputum specimens**,** when MTBDR*plus* and MTBDR*sl* were used sequentially. Out of the 270 sputum specimens collected, 30 and 24 were diagnosed as OFX and AMK mono-resistant, respectively according to phenotypic DST.

**Figure S3:** Diagrammatic representation for the diagnosis of Pre-XDR-TB, defined as OFX mono-resistant (**A**) or AMK mono-resistant (**B**) and XDR-TB (**C**) in the culture isolates**,** when MTBDR*plus* and MTBDR*sl* were used sequentially. From the 270 isolates tested 36, 43 and 86 were determined to be OFX mono-resistant, AMK mono-resistant and XDR-TB, respectively according to phenotypic DST.
